# Comparison of rituximab and conventional adjuvant therapy for pemphigus vulgaris: A retrospective analysis

**DOI:** 10.1371/journal.pone.0198074

**Published:** 2018-09-25

**Authors:** Ashwin Agarwal, Russell P. Hall, Lionel L. Bañez, Adela R. Cardones

**Affiliations:** 1 Department of Dermatology, University of Pennsylvania, Pennsylvania, Philadelphia, United States of America; 2 Department of Dermatology, Duke University Medical Center, Durham, North Carolina, United States of America; 3 Durham VA Medical Center, Durham, North Carolina, United States of America; Van Andel Institute, UNITED STATES

## Abstract

**Background:**

Rituximab is a promising steroid sparing agent used in the treatment of moderate to severe pemphigus vulgaris. Its exact place in the algorithm of pemphigus treatment, vis-à-vis other, conventional adjuvant therapy (CAT) is not known.

**Objective:**

To describe and compare disease course outcomes and morbidity among patients with moderate to severe pemphigus who received rituximab therapy (RT) in addition to prednisone and CAT, versus those who were treated with prednisone and CAT alone.

**Methods:**

A 16-year retrospective case control study was designed with adult patients who were seen at the Duke University Dermatology Immunodermatology clinic from 1999–2015, who had a diagnosis of pemphigus vulgaris, and required prednisone and at least 1 systemic CAT. All patients had at least 6 months follow up from the initial visit. Interventions included RT, systemic CAT, and prednisone. The main outcome measured was prednisone intake. Secondary outcomes were complete remission (CR) and partial remission (PR).

**Results:**

40 patients were included in the study. All initially received prednisone and at least 1 systemic CAT. 13/40 eventually went on to receive RT, while 27/40 remained on CAT (CAT-only). Patients in the RT group, pre-RT, had a median prednisone intake of 658.57 mg/month. Rituximab treatment significantly reduced this to 177.22 mg/month (p = 0.002). Median prednisone intake of the CAT-only group was 141.33 mg/month. This was significantly less than Pre-RT (p = 0.01) and on par with Post-RT intake (p = 0.58). 54% of patients in the RT group and 64% of those in the CAT-only group achieved CR. All patients in the RT group and 96% of those in the CAT-only group achieved at least PR.

**Conclusions:**

32.5% of our patients with moderate to severe pemphigus vulgaris failed prednisone and traditional CAT treatment and required rituximab therapy. Rituximab reduced the monthly prednisone intake in these patients by 73%. This suggests that a subset of patients with moderate to severe pemphigus may benefit from early institution of rituximab therapy. Rituximab significantly reduces the monthly prednisone requirement among CAT-resistant pemphigus vulgaris patients to levels on par with CAT-responsive patients.

## Introduction

Pemphigus vulgaris is mucocutaneous blistering disorder characterized by the presence of pathogenic autoantibodies against desmogleins (Dsg) 1 and 3[1, 2]. It has a chronic, relapsing and remitting course, and in severe cases, can be life threatening. Systemic corticosteroid therapy has dramatically reduced mortality among these patients[3], but can be associated with numerous unwanted side effects [4]^,^ [5].

Treatment of moderate to severe forms of pemphigus vulgaris has relied on the use of steroid sparing agents[6], but the optimal choice for first line steroid sparing adjuvant therapy is still unclear[7]. Randomized, long-term comparative studies are lacking as prospective clinical trials are challenging to perform in this patient population.

Rituximab, a humanized anti-CD20 antibody, has shown great promise in small, non-comparative retrospective studies, non-comparative clinical trials [7–11], and more recently, in a controlled prospective study[12]. Patients experienced rapid control of their disease and re-epithelialization as early as 1–3 months after initiation of therapy, and as many as 58% of patients achieved long term remission[7–10]. A recent consensus statement has recommended rituximab as first line therapy for moderate to severe pemphigus[13].

Herein, we retrospectively compare outcomes among patients with moderate to severe pemphigus vulgaris who received rituximab versus other, more traditional, conventional adjuvant therapy (CAT) at a single institution. In addition, because all patients who received rituximab also received these other adjuvant therapies prior to rituximab therapy (RT), we compare outcomes in these patients before and after their first rituximab dose.

## Methods

### Study patients

We conducted a single-centre retrospective, case-control study of adult (> = 18 years of age at baseline) patients diagnosed with moderate to severe pemphigus vulgaris, who required systemic corticosteroid therapy plus systemic adjuvant therapy, and who were seen at Duke University from May 1999 through February 2015. This study was approved by the Duke University Institutional Review Board, with waiver of consent.

Patients were identified using the Duke Enterprise Data Unified Content Explorer (DEDUCE). Patients were included if they had a clinical diagnosis of pemphigus vulgaris, a direct immunofluorescence (DIF) from perilesional skin that demonstrated keratinocyte cell surface IgG, and at least 6 months of documented clinic follow-up. In addition, patients who received rituximab had to have at least 6 months follow up from their first rituximab infusion.

A total of 68 adult patients with pemphigus vulgaris were identified on initial screening. Among these, 46 patients met the inclusion criteria for age and follow up duration. However, two patients received topical treatment only, and 4 patients received prednisone but did not require systemic adjuvant therapy and were therefore excluded. A total of 40 patients were included in the study. All received oral prednisone and conventional adjuvant therapy (CAT). Thirteen (13) or 32% of these patients went on to receive rituximab (RT group). Twenty-seven (27) or 67.5% of these patients remained on CAT (CAT-only group).

### Clinical and immunologic parameters

Patient demographics, disease duration, and treatment data were collected. All patients received oral prednisone and CAT consisting of mycophenolate mofetil, azathioprine, cyclophosphamide, adalimumab and infliximab. Patients were divided into two groups: (1) those who went on to receive rituximab at any point in the treatment (RT group) and (2) those who did not receive rituximab (CAT-only).

Pemphigus activity was calculated retrospectively using the Pemphigus Disease Activity Index (PDAI) Disease activity score[14–16]. Because information available to calculate the PDAI was limited, and because PDAI has not been validated for retrospective studies, we used this to aid in the description of patients at their initial visit and initial rituximab infusion, but not as an outcome. Complete remission (CR) was defined as the absence of new or old lesions for at least two months on minimal or no therapy. Partial remission (PR) was defined as presence of transient new lesions that heal within one week while the patient was receiving minimal therapy, including topical steroids, or no therapy[15]. Disease status was calculated retrospectively. Minimal therapy was defined as less than, or equal to, 10 mg/day of prednisone (or the equivalent) and/or minimal adjuvant therapy for at least two months. Minimal adjuvant therapy was half of the dose required to be defined as treatment failure, as defined by Murell, et al[15]. Total prednisone intake was manually calculated at each visit, for each patient based on what was documented in the chart. This included any fluctuation in prednisone dosage that occurred in between clinic visits. Monthly prednisone intake was calculated by dividing the total prednisone intake by the number of months in follow up. Number and type of adverse medical events including emergency room visits, hospitalizations, and significant infections were documented and described. Significant infections were defined as any infection requiring systemic (oral or intravenous) antimicrobial therapy. When available, quantitative anti-Dsg1 and anti-Dsg3 antibody titres were collected [17, 18].

Baseline was defined as the first patient presentation to the clinic, regardless of whether they had previously been diagnosed and treated for pemphigus vulgaris. Initial rituximab (IR) treatment was defined as the time at first infusion of rituximab. Among patients who received RT, monthly prednisone intake and serum marker titres were calculated separately based on phase of RT: (1) from baseline to IR (pre-RT), and (2) from IR onwards (post-RT).

Pre-RT and post-RT monthly prednisone intake were compared using Wilcoxon signed-rank test. Monthly prednisone intake of CAT-only patients were compared separately to pre-RT and post-RT prednisone intake of patients in the RT group using crude and adjusted linear regression, controlling for age and gender. Due to non-normal distribution, monthly prednisone intake was log-transformed prior to inclusion in regression models. To describe trends in cumulative prednisone intake, cumulative prednisone intake at each documented follow-up was plotted and the linear slope calculated. T-test was used to compare cumulative prednisone dose at different time points after baseline.

Time to PR/CR, CR or discontinuation of prednisone were compared between the two groups using unadjusted Cox proportional hazards regression. For time dependent outcomes, IR was considered time zero for the RT group. For the CAT-only group, time zero was either the initiation of CAT, or baseline, if the patients were already on CAT during their initial presentation to clinic.

## Results

Thirteen out of the 40 patients, or 32% of the patients included in this study required RT in spite of initial treatment with prednisone and CAT. Twelve of the 13 patients were already on oral prednisone and eight were already on CAT prior to baseline. The median time from baseline to rituximab therapy was 19.6 months (range 0.7 to 89.3; Table 1), and 11 of 13 patients had greater than 6 months follow up from baseline to IR.

**Table 1:** A summary of study patient characteristics in each treatment group including demographics, time to treatment, follow-up data, and previous treatments.

		Treatment Group		
		RT	CAT-only	P value
**Number of Patients, n**	40	13	27	
**Gender**				1.00*
**Male n, (%)**	20	6 (54)	14 (52)	
**Female n, (%)**	20	7 (56)	13 (48)	
**Age in years at Baseline, median (IQR)**	59 (47–67.5)	56 (46–69)	59 (50–67)	0.74
**Disease duration at Baseline in months, median (IQR)**	14.19 (3.12–37.35)	18.92 (8.18–74.48)	10.37 (3.12–33.99)	0.39
**Time from Baseline to first rituximab infusion in months, median (range)**		19.65 (0.66 to 89.3)		
**Follow-up duration in months, median (IQR)**	47.5(27.94–94.04)	60.48 (44.09–87.89)	43 (20–96)	0.50
**Other treatments before Baseline**				
**AZA**		+	+	
**MMF**		+	+	
**MTX**		+	+	
**Cyclophosphamide**		+	+	
**Infliximab**		+	+	
**Dapsone**		+	-	
**Adalimumab**		-	+	
**Prednisone dose at Baseline (mg), median (IQR)**	40 (20–60)	40 (20–60)	40 (15–60)	0.79
**PDAI Score at Baseline, median (IQR)**	15 (10–39.5)	20 (12–44)	15 (5–30)	0.22
**PDAI Score at IR, median (IQR)**		7 (5–19)		0.014****
Anti-Dsg1 at Baseline, median (IQR)**	23.5 (3–89)	44.5 (1.5–146.5)	23.5 (4–78)	0.67
Anti-Dsg3 at Baseline, median (IQR) **	180 (121–421)	186 (127.5–383.5)	180 (99–769)	0.79
Anti-Dsg3 at IR, median (IQR)***		322 (130–552)		0.139****

*Using Fisher’s exact test

**Available in 30 patients, 12 eventually underwent RT

***Available in 10 RT patients

****Compared to Baseline, RT group

### Demographics and patient characteristics

Demographics and patient characteristics are summarized in Table 1. There was no significant difference among the two treatment groups in terms of age, gender or PDAI score at baseline. Median age at baseline for all patients was 59 years (IQR 47–67.5). Median age for RT group was 56 (IQR 46–69), and CAT-only was 59 (IQR 50–67; p = 0.74). Median time from disease onset to baseline or initial clinic visit was higher among the RT compared to the CAT-only group: 18.9 vs 10.4 months, although this was not statistically significant (p = 0.39).

Median PDAI score at baseline overall was 15 (IQR 10–39.5)[16]. Median PDAI score at baseline was not significantly different between the RT group (20; IQR 12–44) and the CAT-only group (15; IQR 5–30; p = 0.22). Median prednisone dose and anti-Dsg3 titer at baseline were similar between the two groups. Median follow-up for all patients was 47.5 months.

### Change in median PDAI and anti-Dsg3 titres from baseline to initial rituximab infusion (IR)

All 13 patients who received rituximab also received at least one other adjuvant therapy prior to rituximab treatment: mycophenolate mofetil, azathioprine, dapsone or infliximab. The median time from baseline to IR was 19.6 months (range 0.7 to 89.3). Each cycle of rituximab treatment was 1 gm/infusion x 2 cycles, 14 days apart. Sixty-nine percent (9/13) of patients who received rituximab received more than one treatment cycle. Five (5) patients received 2 cycles, 2 patients received 3 cycles, 1 received 4, and another 6 cycles. The gap between rituximab cycles was at least 12 months, although one patient had a six-month gap between his first and second cycle.

Eleven patients in the RT group had at least six months follow up between their baseline or initial clinic visit and IR. Among these patients, the median PDAI score at IR was half of the median PDAI at baseline (baseline = 15, IR = 7; p = 0.014; Table 2). Median anti-Dsg3 titre increased from 147 at baseline (IQR 126–244) to 322 (IQR 130–552) at IR, although this was not statistically significant (p = 0.14).

**Table 2:** Outcomes among pemphigus patients who went on to receive rituximab vs. those who stayed on conventional adjuvant therapy.

	Treatment Group		P value
	RT	CAT-ONLY	
**Number of patients who discontinued prednisone, n(%)**	8 (62)	17 (63)	1.00
**Time to discontinue prednisone in months, median (IQR)**	14.31 (10–22.29)	11.04 (7.23–16.33)	0.38
**Achieved complete remission n(%)**	7 (54)	17(63)	0.98
**Time to first complete remission in months, median (IQR)**	6.05 (5.13–7.52)	10.74 (5.06–24.60)	0.84
**Achieved at least partial remission n(%)**	13 (100)	26 (96)	0.49

### Reduction in monthly prednisone intake

**Fig 1:**
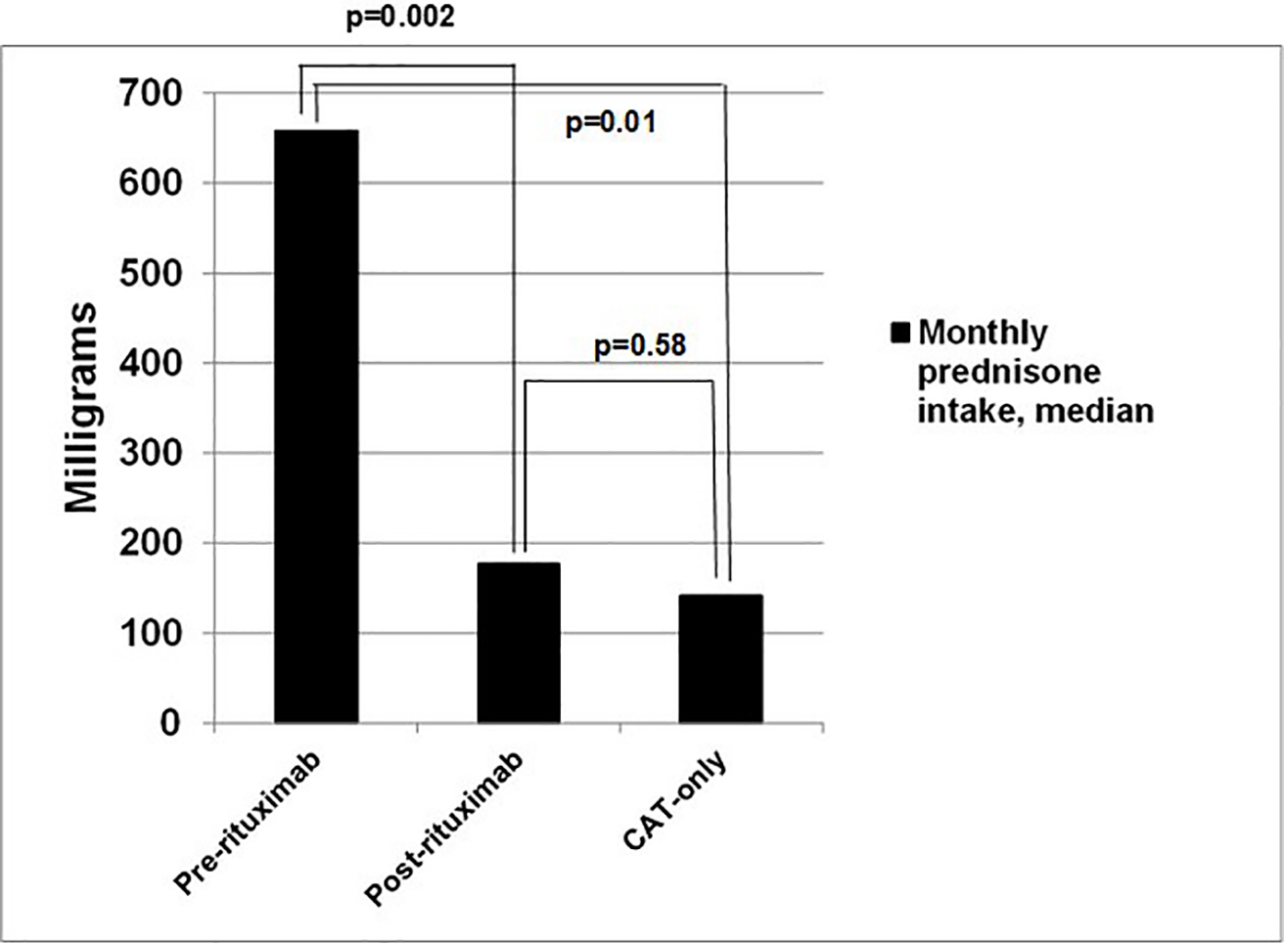
Median prednisone dose among RT patients in their pre and post rituximab phases, and CAT-only patients.

In the RT group, the pre-RT median monthly prednisone intake was 658.6 mg/month (IQR 600–724.8), but only 177.2 mg (IQR 97.3–309.5) post-RT (p = 0.002; Fig 1). Patients in the CAT-only group had significantly lower monthly prednisone intake (141.3 mg, IQR 79.3–428.8) compared to pre-RT patients, in both crude (p = 0.02) and adjusted (p = 0.01) regression models. However, there was no difference in monthly prednisone intake between CAT-only and post-RT patients (p = 0.58) even after adjusting for age and gender (p = 0.61).

**Fig 2:**
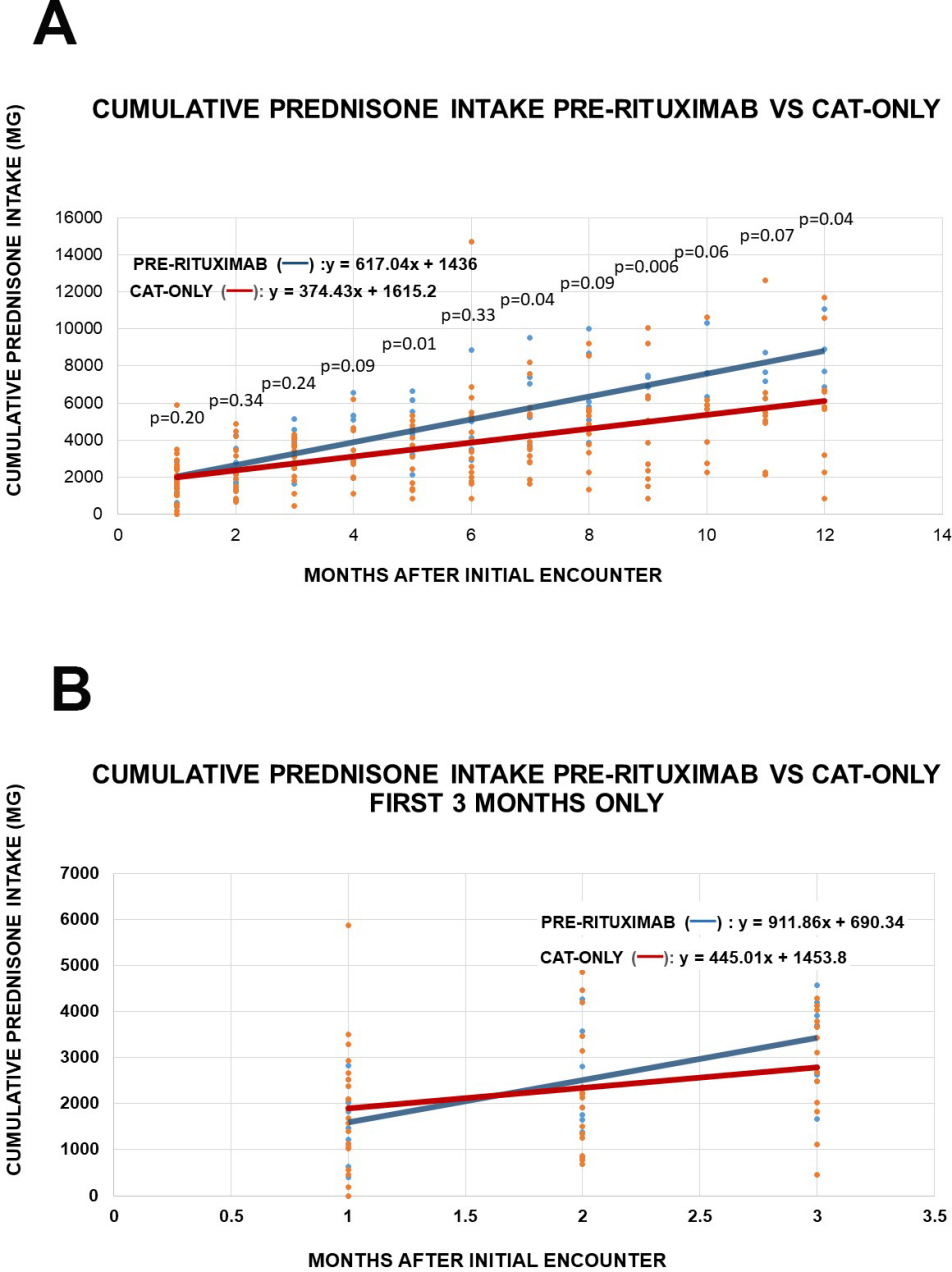
**(A).** Cumulative prednisone intake among patients who received prednisone plus CAT who either failed and went on to receive rituximab (PRE-RITUXIMAB) or successfully remained on CAT only (CAT-only). Data graph and linear slope over 12 months (A), or the first 3 months only **(B)**.

RT patients had varying durations of their pre-RT phase. During the pre-RT phase, the rate of increase in cumulative prednisone intake was greater in pre-RT patients compared to CAT-ONLY patients (Fig 2A). The calculated slope at 3 months estimates the monthly prednisone intake to be 911 mg for patients in the RT group (pre-RT) and 445 mg for the CAT-only group (Fig 2B).

### Hospitalization rates, infections and other outcomes

**Fig 3:**
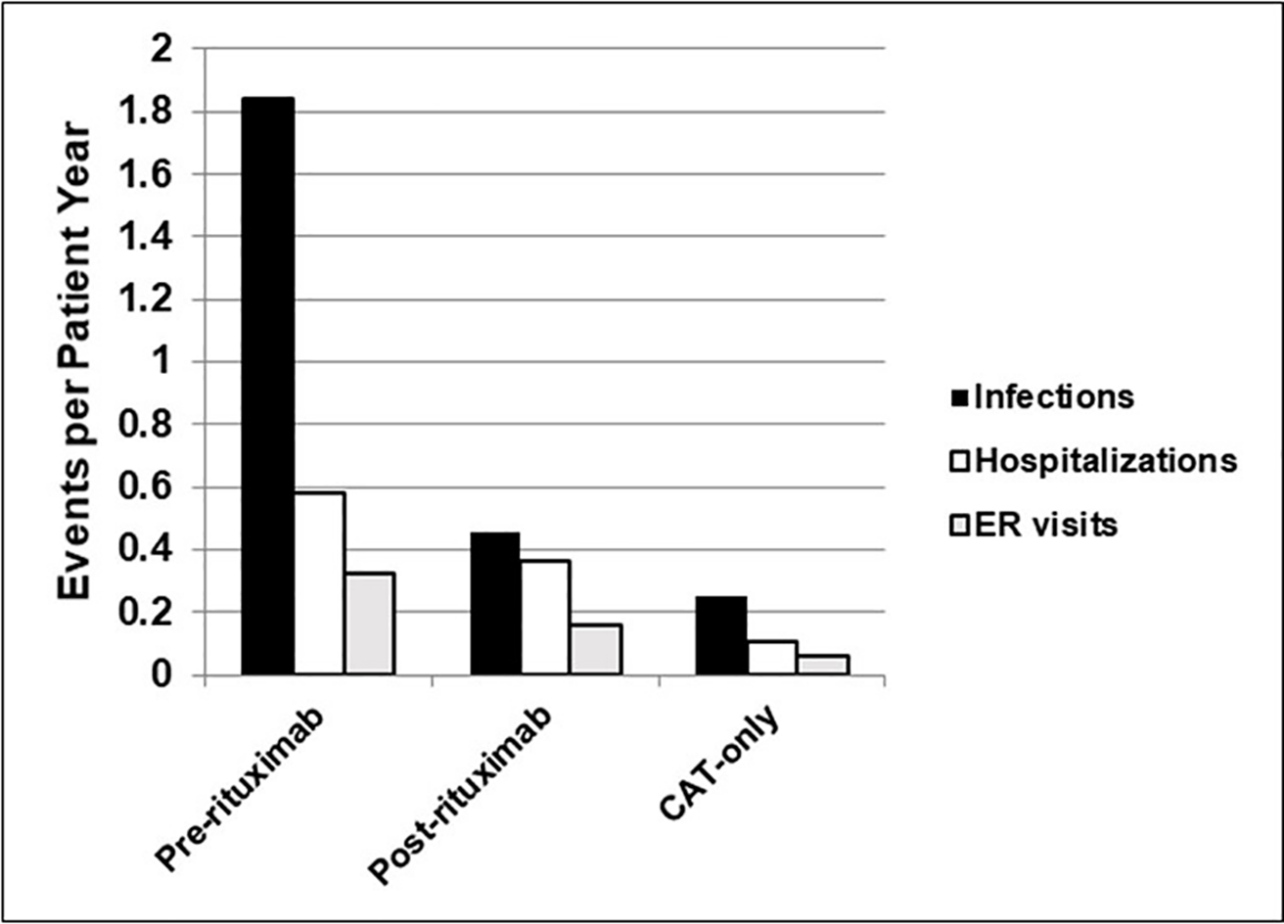
Number of infections requiring oral or IV therapy, hospitalization and ER visits, per patient year, among RT patients (pre and post-rituximab) and CAT-only.

The number of infections, hospital admissions and emergency room (ER) visits per patient year was higher in the pre-RT phase. Among RT patients in their pre-RT phase, there were 51 episodes of infectious events requiring either oral or IV antibiotic therapy, 16 hospital admissions and 9 ER visits over 332 patient months (1.8/patient year, 0.6/patient year and 0.3/patient year, respectively; Fig 3). Post-RT however, there were only 20 episodes of significant infection events, 16 hospital admissions and 7 ER visits over 524 patient months (0.5/patient year, 0.4/patient year and 0.2 per patient year respectively). Patients in the CAT-only group had only 33 significant infections, 14 hospital admissions and 8 ER visits in 1565 patient months (0.3/patient year, 0.1/patient year and 0.1/per patient year, respectively).

A summary of other outcomes is listed in Table 2. There was no difference in the likelihood of complete remission (CR) [hazard ratio (HR) 1.01; 95% confidence interval (CI: 0.43–2.67); p = 0.98] among RT patients compared to the CAT-only patients. Among patients who went into CR, RT patients had a shorter median time to remission (6.0 months) compared to CAT-only patients (10.7 months). However, this was not statistically significant. All patients except one in the CAT-only group achieved at least PR. Seven (54%) patients in the RT group achieved CR compared to 18 (64%) patients in the CAT-only group. There was no difference in time to PR/CR between the two groups [HR 0.78; 95% CI (0.39–1.57); p = 0.49]. Finally, there was no difference in the likelihood of discontinuation of prednisone [HR 0.93; 95% CI (0.39–2.21); p = 0.87] among RT patients compared to the CAT-ONLY patients.

## Discussion

Rituximab has emerged as one of the most promising targeted therapies for moderate to severe pemphigus vulgaris, inducing clinical remission as early as 1–3 months after initiation of treatment[7, 8]. Although it had been used previously as second or even third line therapy, current recommendations include rituximab as first line therapy for patients with moderate to severe pemphigus. [13, 19]. Indeed, patients treated with rituximab were more likely to go into CR, and had less severe side effects compared to those who received systemic corticosteroid therapy[20].

This study focuses on a subset of pemphigus patients who required adjuvant therapy in addition to prednisone. A third of the patients in this study clearly were adjudicated to have failed CAT therapy by their clinicians and were given rituximab (RT group). These patients had a drop in their PDAI score from their initial visit to the time they received rituximab, likely due to prednisone. Their anti-Dsg3 titres, on the other hand, were increasing, although we did not have enough patients in our study to achieve statistical significance. Those in the RT group also had a median disease duration 1.8 times longer before their initial visit to our specialty dermatology clinic. Since retrospective data suggests that those who receive rituximab earlier in the course of their disease may respond more quickly [9], the importance of identifying this subset of patients who would do best on rituximab earlier in the course of their disease is evident.

Monthly prednisone requirement distinguishes this CAT-resistant, RT group from those who stayed on CAT alone (CAT-only). The monthly prednisone requirement in the pre-RT group was almost four times that of the CAT-only group in both crude (p = 0.02) and adjusted (p = 0.01) regression models. Based on our estimates at the 3-month time point, our RT patients were accumulating around 911 mg of prednisone per month (around 30 mg per day) in their pre-RT phase vs. 445 mg per month (around 14.8 mg per day) in the CAT-only group. Rituximab significantly decreased the monthly prednisone intake to match levels in CAT-sensitive patients (p = 0.58), even after adjusting for age and gender (p = 0.61). Although on the surface, the likelihood of remission was not significantly different between the two groups, RT patients had more severe disease, as reflected by their increasing requirement for prednisone and anti-Dsg3 titers in spite of prednisone and CAT therapy. This suggests that a subset of patients with moderate to severe pemphigus would benefit maximally from early institution of rituximab, and this subset can be identified by requirement for significant prednisone (30 mg per day) and increasing anti-Dsg titers.

The pre-RT incidence of infections, hospitalizations and ED visits was also markedly higher in the RT group compared to CAT-only patients, and improved after institution of RT. This suggests that a subset of patients with moderate to severe pemphigus would benefit maximally from early institution of rituximab. Our data suggests that this subset can be identified after 3 months or less of CAT therapy by the requirement for 30 mg of prednisone or more and increasing anti DSG titers.

In summary, rituximab may facilitate remission by a different, more efficient mechanism compared to CAT as it decreased prednisone intake dramatically and significantly in the RT group despite their more severe disease. These patients, who comprised 33% of our pemphigus cohort, may present to clinic with similar demographics and PDAI scores as those who respond to conventional adjuvant therapy. This study proposes the use of monthly prednisone intake and anti-Dsg3 titre trends as quantitative metrics that can be measured within the first three months of therapy to identify the patient subset that may benefit most from early rituximab therapy. For these CAT non-responders, early identification and intervention can decrease the duration of active disease, total prednisone intake and associated comorbidities, and adverse events including hospitalizations and systemic infections. The retrospective nature of this study and small sample size given the rarity of this disease warrant prospective validation with larger comparative prospective studies comparing CAT-only therapy with rituximab therapy. In addition, the use of 10 mg/day of prednisone or lower to define minimal therapy may limit our estimation of prednisone-associated adverse effects, as even low dose corticosteroid therapy can be associated with increased blood pressure, Cushingoid changes, and weight gain, among others[21]

It is possible that even CAT responders may also benefit from earlier rituximab intervention in terms of fewer infections, hospitalizations, lab draws, and clinic visits. Further, other outcome measures such as quality of life and cost need to be further studied in prospective studies and may suggest additional benefit of rituximab therapy in both CAT-responsive and CAT-resistant patients. It is important to recognize these two subsets of CAT-responsive and CAT-resistant patients in designing these clinical trials to elucidate rituximab-specific benefits.
